# A Case of Douloureux of Twelve Years’ Standing Treated by Large Doses of the Salicylates, with Marked Success

**Published:** 1887-11

**Authors:** Francis X. Dercum


[TO BE CONTINUED.]

---

### A CASE OF TIC DOULOUREUX OF TWELVE YEARS' STANDING TREATED BY LARGE DOSES OF THE SALICYLATES, WITH MARKED SUCCESS.

BY FRANCIS X. DERCUM, M. D.

On November 30th, 1886, I was summoned to attend Mrs. K., aged 52 years, a German by birth, of good physique, and mother of a number of healthy children. She was a sufferer from tic douloureux. As she spoke she was constantly interrupted by frightful paroxysms of pain. She moved her jaws as little as possible, and her words were uttered so cautiously as at times to be indistinct. She gave me her history with great difficulty and at the expense of much suffering. Twelve years ago, while apparently in good health, she was suddenly attacked by pains in the right side of the face. These gradually increased in intensity. Previous to the onset she had no illness whatever, and she had not as yet entered upon the menopause. All of her functions, as far as I could ascertain, had been normal. She herself attributed her affection to an attack of cold.

Her suffering had been practically continuous. The paroxysms had varied at times both in frequency and severity. Sometimes they occurred many times a minute, and again but four or five times an hour. When their intensity was at its greatest height, she felt frightful darting pains, which culminated—to use her own expression—as though her face were being rent to pieces, or as though it were bursting from an explosion. When the pains were comparatively mild, they were simply shooting or darting in character. She was always worse in cold, damp, or stormy weather, and suffered much less when the weather was mild and clear.

A close examination revealed the fact that the nerve-distribution most affected was that of the superior maxillary. The inferior maxillary was also decidedly affected, but the opthalmic division did not seem to be affected at all. The cheek, especially about the infra-orbital and malar regions, was excessively hyperæsthetic. A slight touch was sufficient to provoke a spasm of pain. The same was true, though to a less extent of the gums—*i. e.*, on the affected side—and from which the bulk of the teeth, the molars and premolars, had been fruitlessly removed.

Further questioning elicited the statement—and it was an all-important point in her history—that during the entire period of twelve years she had but one remission—*i. e.*, one *complete* remission,—and this had occurred under the following circumstances: Some two years ago her physician had ordered her to Atlantic City. Here she contracted severe rheumatism, which affected mainly the lower extremities. The attack lasted upward of eight weeks, and during all of this time she was free from her tic douleureux. Just as soon, however, as the rheumatism subsided her tic reappeared, and had continued with varying degrees of intensity up to the present time.

Notwithstanding the history of a prolonged remission, I hesitated about giving the patient any encouragement. She had been under the hands of various physicians, who had made unusual efforts in her behalf, and who had finally pronounced her case incurable. I, too, told her that we could expect but little from drugs, and that the only remedy that suggested itself to me was a surgical procedure, the propriety and results of which were themselves open to questions.

Two views, of course, presented themselves regarding this case; one that it was of organic origin, the other that it was symptomatic of some general disease. In favor of the first view was the long-continued existence of the affection. The latter had existed for twelve years, but it appeared to me that the history of a marked and prolonged remission of eight weeks rendered an organic origin extremely improbable. Certainly we should not expect a diseased bone or membrane to accord with such a history. On the other hand it was difficult to accept the second view. The patient presented no evidence of constitutional trouble. Her general health, as it so often is in these cases, was good. There was no evidence of specific disease, and no affection of the kidneys. The patient's history suggested a doubtful connection of rheumatism with the trouble, but it seemed strange that, if rheumatic, the latter should have persisted for so many years.

I finally decided, before attempting the operation of nerve-stretching, to test the question of rheumatism very thoroughly; and, accordingly, at my next visit prescribed oil of gaultheria in fifteen-minim doses, to be taken every three hours. After four days had elapsed I called again, and, to my surprise and pleasure, found that my patient was decidedly better. The paroxisms were far less frequent and much less painful. The hyperæsthesia of the cheek and gums had also together disappeared. The physiological action of the salicylate was evidenced by decided tinnitus. I directed the remedy to be continued, and called at intervals of three or four days until December 20th, when I found my patient suffering from a diarrhœa, the result of an indiscretion in diet. Up to this time she had steadily improved, and she was to all intents and purposes well. Previously she had never been able to eat without exciting pain. The pleasure of eating with comfort had resulted in eating too much, and hence the diarrhœa.

I now stopped the oil of gaultheria, and for a number of days directed my attention to her digestion. The diarrhœa was promptly relieved, and on December 24 she ventured out of the house. It was early in the morning, there was snow upon the gronnd, and it was very cold. This exposure was followed by a mild recurrence of the tic. She had a few paroxyisms of moderate severity. She, of her own accord, resumed the oil of gaultheria.

On December 26th I saw her again, and now replaced the oil of gaultheria by sodium salicylate, of which I gave twenty grains every four hours. This she tolerated well. Every trace of the douloureux now disappeared. She voluntarily increased the dose of the salicylate to thirty grains, thus taking one hundred and twenty grains a day. Her fear of a return of her terrible affliction caused her to persist in this excessive dose until December 29th,, when, upon my urgent advice, she diminished the dose to twenty grains three times a day. The large doses had caused marked depression, her tinnitus was excessive, she was decidedly deaf, and I fancied that her pulse was weak. Besides, she was taking very little food, and her digestion was much impaired. Although she had not the slightest return of pain at any time, she persisted in the use of the salicylate until January 12th, when it was finally abandoned.

Since that time she has been exposed to the weather on numerous occasions and at various hours of the day and evening. Upon one occasion, indeed, she caught a severe cold, which gave rise to a severe bronchitis, but without any return of the douloureux. At present writing she is entirely well. How long she will continue so is, I confess, a matter of doubt. However, I believe that the present cessation of pain is directly due to the action of the salicylate, and to nothing else. The effect was too sudden and striking to be attributed to other causes. When the oil of gaultheria was stopped on account of the intercurrent diarrhœa, the pain reappeared, but immediately ceased when the salicylate was resumed. I believe also that the good effects of the drug were due to the large doses employed.

The mode of action of the salicylates in th s case is a matter of interesting speculation. It is possible, of course, that the affection was rheumatic in its origin, and that the salicylates were efficient on this account. On the other hand, it is not impossible that the remedy acted in another and entirely different way, and the following hypothesis seems to me plausible. That salicylic acid acts directly on the nervous system cannot, I think, be doubted. To me the single fact of the ringing in the ears is an evidence of such action, and the further fact that increased doses result in deafness a confirmation of this idea. Now, if salicylic acid produces ringing in the ears, and even deafness, it is probably by a direct action of the drug on what we may call the acoustic protroplasm. That is, the acoustic protroplasm is *molecularly impressed*; it has the rates and directions of its molecules so affected as to give rise first to abnormal sensations, and finally it has the molecules so much arrested or inhabited as to give rise to an entire loss of function. May it not be that the action ot the salicylate in this case is due to its modifying impress on the abnormal movements going on in the molecules of the diseased lung? and is it not possible that it deadened the hyperæsthesia and annulled the pain by virtue of the same quality by which it produces deafness? I give this speculation for what it is worth.

My hope in reporting this case is that others will give the salicylates a fair trial, even in cases in which there is no rheumatic history; and further, that in making this trial they will steadily increase the dose until an effect is produced or the limits of safety are reached.—*The Polyclinic.*

